# Improved Retinex algorithm for low illumination image enhancement in the chemical plant area

**DOI:** 10.1038/s41598-023-48664-7

**Published:** 2023-12-11

**Authors:** Xin Wang, Shaolin Hu, Jichao Li

**Affiliations:** 1 School of Electronic Information Engineering, Xi’an Technological University, Xi’an, 710021 China; 2 School of Automation, Guangdong University of Petrochemical Technology, Maoming, 527523 China

**Keywords:** Electrical and electronic engineering, Computer science

## Abstract

Due to the complexity of the chemical plant area at night and the harsh lighting environment, the images obtained by monitoring equipment have issues such as blurred details and insufficient contrast, which is not conducive to the subsequent target detection work. A low illumination image enhancement model based on an improved Retinex algorithm is proposed to address the above issues. The model consists of a decomposition network, an adjustment network, and a reconstruction network. In the decomposition network, a new decomposition network USD-Net is established based on U-Net, which decomposes the original image into illumination and reflection maps, enhancing the extraction of image details and low-frequency information; Using an adjustment network to enhance the decomposed lighting image, and introducing a Mobilenetv3 lightweight network and residual structure to simplify the network model and improve the contrast of the image; In the reconstruction network, the BM3D method is used for image denoising to enhance the ability to restore image detail features; The enhanced illumination and reflection images were fused based on the Retinex algorithm to achieve low illumination image enhancement in the chemical plant area. This article uses five image quality evaluation indicators, namely Peak Signal-to-Noise Ratio, Structural Similarity Index, Natural Image Quality Evaluator, Interpolation Error, and Level of Effort, to compare eight traditional or modern algorithms and evaluate three different types of datasets. The experimental results show that the improved algorithm enhances the texture details of the image, improves the contrast and saturation of the image, and has good stability and robustness, which can effectively meet the needs of low illumination image enhancement in chemical plant areas.

## Introduction

Safety problem has always been the most important problem in the field of chemical production. Due to the processing and production in the chemical plant area, the plant area is full of dangerous factors. Therefore, the plant area not only bans fire but also strictly controls the personnel and vehicles. Staff needs to wear personal protective equipment such as helmets at all times. In order to prevent the occurrence of such accidents, 24-hour intelligent monitoring is indispensable. However, the arrival of the night will make the details of the image ignored due to insufficient light. Features, which lead to a series of safety accidents^1,2^. At present, many intelligent monitoring systems use infrared thermal imaging equipment to detect abnormal accidents, but it is only suitable for scenes with sufficient light, and the cost is high, which makes it easy to cause a waste of resources^3^. Therefore, this paper uses the improved Retinex algorithm to enhance the low illumination of the target image and uses the lower cost to make the low illumination image achieve better image quality, and further realize the safety detection of chemical plant areas.

In recent years, with the continuous development of deep learning, some scholars have applied it to low-light image enhancement and achieved excellent results. Aiming at the problems of low brightness, obvious noise, poor contrast, and difficulty in obtaining dark area details in a low-light environment. Sun et al.^4^ proposed to combine the improved particle swarm optimization algorithm with the single-scale Retinex algorithm, but the processing speed was slow, ignoring the problem of the number of model parameters; Wei et al.^5^ proposed a deep Retinex-Net algorithm for learning on LOL datasets, but there are problems such as excessive noise; Dong et al.^6^ described a novel and effective low-light video enhancement algorithm. The algorithm reversed the input low-light video and applied the dehazing method to the reversed image. The algorithm has obvious improvement in quality and speed, but the enhanced image has some distortion; Guo et al.^7^ realized low illumination enhancement by estimating only the illumination map of the low illumination image, which greatly reduced the solution space and reduced the amount of calculation, but ignored the processing of the reflection map, resulting in over-sharpening of the enhanced image; Zhang et al.^8^ established a simple and effective ‘lighting dark’ network-KinD-Net. The network has strong robustness for serious visual defects and has strong applicability. It can adjust the illumination level arbitrarily, but the artificially set parameters are random, and it will be difficult to manually select the appropriate parameters during verification or practical application.

Therefore, aiming at the problem of network model redundancy and unsatisfactory enhancement effect, this paper proposes a low-light image enhancement model based on an improved Retinex algorithm and verifies the feasibility of the model through experiments. The basic structure of this paper is the first section introduces the Retinex model and the improvement of each module; the second section describes the analysis and verification of the experiment. Eight advanced low-light enhancement algorithms are used to carry out comparative experiments and analyze the experimental results. The third section is the summary and summary of the full text.

## Model architecture with modles’ modification

### Overall structure

**Figure 1:**
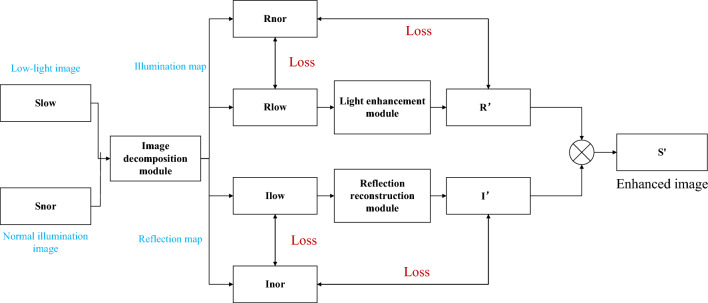
Overall structure.

Due to the complexity of the chemical plant area, it is less difficult to detect targets by routine monitoring during the day. Still, at night, the difficulty of target detection will be doubled due to the concealment of image details. To accurately detect personnel and vehicles, the detail recovery and feature enhancement of low illumination images are necessary. Therefore, given the above problems, this paper constructs an image processing model as shown in Fig. 1. This model mainly comprises a decomposition module, illumination enhancement module, reflection reconstruction module, and target detection module. The image in the chemical plant area is input into the image decomposition module and the low illumination image is decomposed into an illumination image and reflection image. The brightness adjustment and feature enhancement processing are carried out in the illumination enhancement module and the reflection reconstruction module. The processed illumination image and reflection image are fused based on the Retinex algorithm^9^.

The Retinex algorithm divides the image into the illumination component and a reflection component, as shown in formula (1):

1 $$\begin{aligned} F(x,y) = R(x,y)*L(x,y) \end{aligned}$$

In formula (1), (x,y) is the coordinate corresponding to the two-dimensional element, F(x,y) is the image component, R(x,y) is the reflection component, and L(x,y) is the illumination component. The reflection component reflects the essence of the thing. The Retinex algorithm removes the illumination component from the image component and displays the essential information of the image.

### Image decomposition module

**Figure 2:**
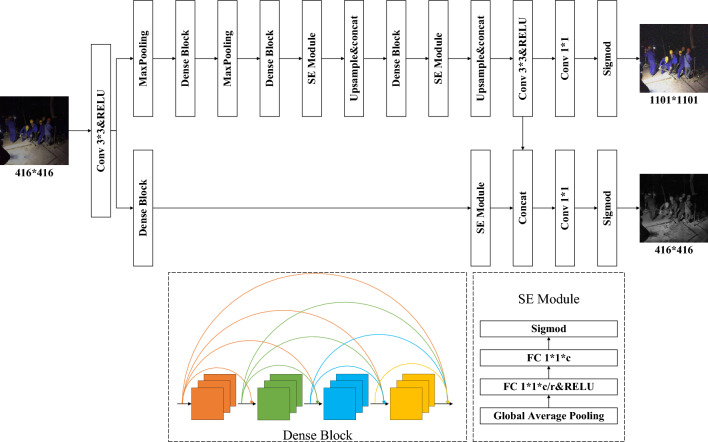
Decomposition network structure.

In the image decomposition module, this article establishes a USD-Net based on the classic U-Net^10^ network structure, which can effectively extract the feature information of the image. Its most prominent features are the symmetrical U-shaped structure and jumping connections. This special structure can better extract illumination and reflection images from pictures. After the top-level pooling layer and convolution layer in the network, the nonlinear activation function RELU is used to simplify the calculation process further and reduce the calculation amount of the model. Due to the small number of layers and parameters in the U-Net network, it is easy to experience deficiencies during the training process. Therefore, to increase the parameters of the network model, dense blocks^11^ were replaced with ordinary convolutional modules to enhance the generalization ability of the network model. At the same time, to strengthen the model’s attention to essential features and reduce the extraction of unnecessary features, this article adds an SE Net channel attention mechanism^12^ based on the U-Net network structure, so that the model always pays attention to essential features during the training process, thereby improving the accuracy and robustness of the decomposition network. The specific network structure is shown in Fig. 2.

The USD-Net takes the low-illumination image $S_{low}$ and the normal-illumination image $S_{nor}$ as input and estimates the illumination component $I_{low}$ and the reflection component $R_{low}$ of $S_{low}$, and the illumination component $I_{nor}$ and the reflection component $R_{nor}$ of $S_{nor}$, respectively. Firstly, the image feature information is extracted through the 3*3 convolution layer, and the upper calculation amount is reduced through the maximum pooling layer. The RGB image is mapped to the illumination component I and the reflection component T by the RELU activation function and the convolution layer. Finally, R and I are normalized to [0,1] by the sigmod function.

According to the Retinex algorithm, the change of incident light will not affect the inherent properties of the object to light, so the decomposed reflectivity $[{R_{low}},{R_{nor}}]$ should be consistent. The specific formula can be expressed as :

2 $$\begin{aligned} L_{rs}^{D} = {\left\| {{R_{low}} - {R_{nor}}} \right\| _1} \end{aligned}$$

$L_{rs}^{D}$ is the similarity of the normalized reflection components, ${\left\| \cdot \right\| _1}$ and is the ${l_1}$ loss.

The second part is the smoothing consistency loss $L_{is}^D$ of the decomposed illumination map. The penalty for this loss on the edge position is minimal, and the liability on the flat area will be relatively large. For example, the structure of formula (3) will minimize the risk of excessive smoothing of illumination.

3 $$\begin{aligned} L_{is}^D = {\left\| {\frac{{\nabla {I_{low}}}}{{\max \left( |\nabla {S_{low}}|,\varepsilon \right) }}} \right\| _1} + {\left\| {\frac{{\nabla {I_{nor}}}}{{\max \left( |\nabla {S_{nor}}|,\varepsilon \right) }}} \right\| _1} \end{aligned}$$

In the formula,$\nabla$ represents the gradient along x (horizontal direction) and y (vertical direction), S is the input image, and $\varepsilon$ is a smaller constant, mainly to avoid being divided by 0.

Since the paired images used in this paper are low-illumination images and the corresponding normal illumination images, when the gradient difference between the two is large or small, it indicates that the illumination at this time is on the surface or edge of the balanced object. Only when the gradients are similar, the penalty is performed. The loss function is the mutual consistency loss $L_{mc}^{D}$ , which is specifically expressed as :

4 $$\begin{aligned} L_{mc}^{D} = {\left\| {M \otimes \exp ( - c \cdot M)} \right\| _1} \end{aligned}$$

Where M is the sum of $\nabla {I_{low}}$ and $\nabla {I_{nor}}$, and c is a constant.

The fourth part is the reconstruction error function $L_{re}^D$, which restricts the reflection and illumination components generated by the decomposition to be as consistent as possible after reconstruction and before decomposition.

5 $$\begin{aligned} L_{re}^D = {\left\| {{S_{low}} - {R_{low}} \otimes {I_{low}}} \right\| _1} + {\left\| {{S_{nor}} - {R_{nor}} \otimes {I_{nor}}} \right\| _1} \end{aligned}$$

Among them, ${S_{low}}$ and ${S_{nor}}$ represent the input low illumination and normal illumination images, while ${R_{low}}$, ${R_{nor}}$, ${I_{low}}$, and ${I_{nor}}$ represent the reflection and illumination components of the decomposed low illumination and normal illumination.

Therefore, the total loss function ${L^D}$ of this module consists of four parts:

6 $$\begin{aligned} {L^{D}} = L_{re}^{D} + {\lambda _{rs}}L_{rs}^{D} + {\lambda _{is}}L_{is}^{D} + {\lambda _{mc}}L_{mc}^{D} \end{aligned}$$

Among them, ${\lambda _{rs}}$, ${\lambda _{is}}$ and ${\lambda _{ms}}$ represent the similarity, smooth consistency, and mutual consistency loss coefficients of the normalized reflection components, respectively.

### Light enhancement module

In this paper, MobileNet-v3 lightweight network^13^ is used in the network structure of the illumination enhancement module, which has fewer parameters and calculations than v1 and v2 network structures. This article conducts network complexity experiments on Mobilenetv1, v2, and v3, respectively. The parameter quantity, computation quantity, and delay time of the network model are selected as the evaluation indicators of model complexity, as shown in Table 1.

**Table 1:** Complexity comparison of MobileNet series.

Network	Params (M)	FLOPs (B)	Latency (ms)
MobileNetv1	5.1	2.6	270
MobileNetv2	4.3	1.6	200
MobileNetv3	**2.49**	**0.42**	**67.2**

The best-performing values are in bold.

**Figure 3:**
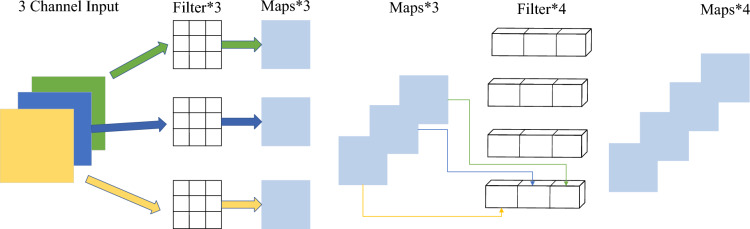
Deep separable convolution structure.

By analyzing the data in Table 1, it can be seen that MobileNetv3 has achieved the lowest parameter count, computational complexity, and latency. Therefore, this article selects v3 as the network structure of the lighting enhancement module. The network reduces the computational complexity of the network by utilizing deep separable convolution. It uses different convolution kernels for each input channel, that is, the number of packets in the network is equal to the number of channels in the network, to reduce the computational complexity of the network as much as possible, which can be divided into two processes: a. The channel direction channel can be separated into convolutions; b. Normal 1*1 convolution outputs the specified number of channels. The specific structure is shown in Fig. 3.

**Figure 4:**
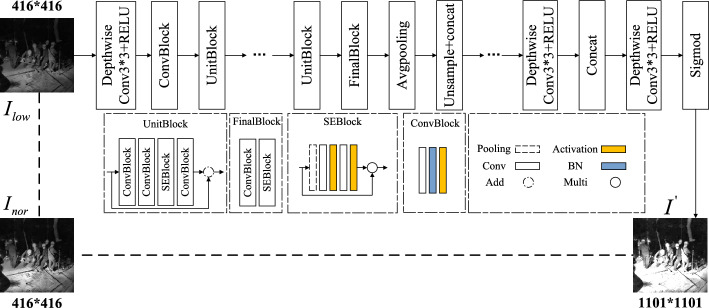
Illumination enhancement network structure.

The illumination enhancement network is shown in Fig. 4. The low-illumination image is input into the feature extraction network with MobileNet-v3 as the main body. Compared with the ordinary convolution network, the separable convolution layer and channel attention mechanism are added, which significantly deepens the depth of extracting image features. ConvBlock consists of a convolution function, a batch normalization function, and an activation function. UnitBlock consists of three ConvBlocks and one SEBlock. The residual structure is used to avoid the complexity of the model to a certain extent. The pooling layer uses an average pooling layer to convert the input feature map into 1*1.

The loss function designed by this module is as follows:

7 $$\begin{aligned} {L^I} = \left\| {{I^{'}} - {I_{nor}}} \right\| _2^{2} + \left\| {|\nabla {I^{'}}| - |\nabla {I_{nor}}|} \right\| _2^{2} \end{aligned}$$

In the formula, ${I^{'}}$ is the illumination image of the illumination enhancement module, and ${I_{nor}}$ is the target image, which ensures the similarity between the adjusted illumination image and the normal illumination image.

### Reflection reconstruction module

In the image decomposition module, the smooth consistency loss is used for the illumination image. When the illumination image is as smooth as possible, the details will be retained in the reflected image. The biggest problem is the noise generated by the reflected image^14^. The existence of noise will seriously affect the quality of the reflected image. To better eliminate the influence of noise, this paper introduces a non-local denoising method–BM3D algorithm^15^. This image-denoising algorithm is based on traditional methods proposed by Kosadin, Alessandro, et al. from the Tampere University of Technology in Finland in 2007. Compared with other algorithms, BM3D currently has the best denoising performance among non-AI images, this algorithm can preserve image feature information to the maximum extent to meet the subsequent image fusion work.

**Figure 5:**
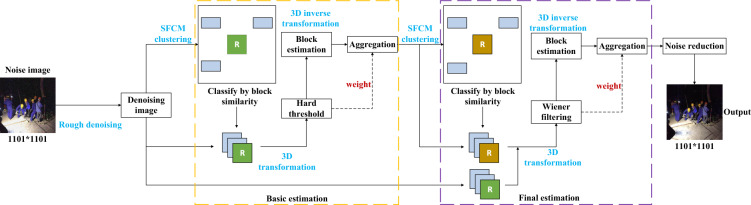
BM3D algorithm flow chart.

The denoising process of the BM3D algorithm is mainly divided into two steps, namely, basic estimation and final estimation. In the basic estimation stage, the image is first subjected to rough denoising, and then the processed image is divided into many small blocks, and the similar blocks in the whole image are searched. The small blocks with high similarity are mixed from two dimensions into three-dimensional heaps, and a 3D transform is performed on them. The intrinsic signal and the separated noise can be obtained in the transform domain and then truncated by hard threshold filtering to initially remove the noise, but the three-dimensional heap needs to be restored to the image by 3D inverse transform. In the final estimation stage, the remaining steps are basically the same except that the threshold is not needed. The filtering is changed to Wiener filtering. Figure 5 is the algorithm flow chart of BM3D denoising.

To verify the feasibility of the BM3D algorithm, the non-reference image evaluation index NIQE (Natural Image Quality Evaluator) was selected as the evaluation standard for image quality before and after denoising, and its numerical value was negatively correlated with image quality.

**Table 2:** Comparison results of NIQE after denoising using the BM3D algorithm.

Type	Original	BM3D
People	5.45	3.73
Car	5.51	3.84
Factory	5.73	3.54

According to Table 2, the NIQE values of the BM3D algorithm after denoising are lower than those of the original input noisy image, with an average NIQE value of 3.7, indicating better naturalness and image quality.

## Experimental verification

### Experimental environment

The experiment of the improved Retinex low-light image enhancement model is completed under the Windows11 operating system, based on the framework of Tensorflow 1.5.0; the GPU is NVIDIA GeForce RTX 3060 Laptop GPU, memory 16GB; the CPU is Intel (R) Core (TM) i7-12700H.

In the experiment, each module was trained separately, and each module was trained using an Adam optimizer with a learning rate set to 10e−6. The number of training sessions for the decomposition network is set to 1000, and the weight parameters in references^5^ and^16^ are set to ${\lambda _{rs}} = 0.01$, ${\lambda _{is}} = 0.08$, and ${\lambda _{mc}} = 0.1$; The resolution of low light image input in the lighting enhancement network is 416 * 416, and the number of training rounds is set to 1000, batch_size is set to 16, patch_size is set to 48 * 48, eval_every_epoch is set to 100.

### Comparison of low illumination image enhancement effects in chemical plant areas

To verify the feasibility and practicability of the model in this paper, the most advanced algorithm is compared with the improved algorithm^17^. The selected algorithms include LIME, Retinex-net, AINDANE(Adaptive and integrated neighborhood-dependent approach for nonlinear enhancement of color images)^18^, StableLLVE(Learning Temporal Consistency for Low Light Video Enhancement From Single Images)^19^, Demist enhancement^20^, BIMEF(A Bio-Inspired Multi-Exposure Fusion Framework for Low-light Image Enhancement)^21^, GLAD(Low-light enhancement network with global awareness)^22^ and ISP(Image Signal Processor)^23^.

**Figure 6:**
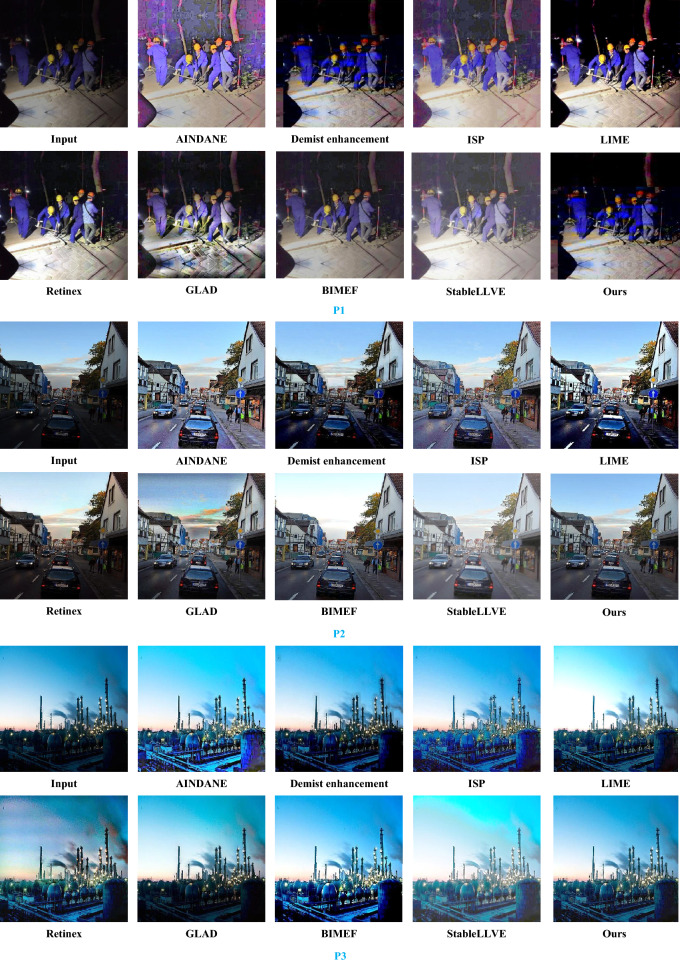
Different algorithms enhance the effect diagram.

Figure 6 shows the effect of different algorithms in enhancing low-illumination images. P1, P2, and P3 in the figure represent three different types of factory images, with P1 representing factory workers working at night; Due to the lack of a low illumination data set of Tank trucks, P2 selects the image of vehicles in the LOL data set. The image contains multiple vehicles and buildings, this image features more information, which is more complex than a simple Tank truck. Therefore, this image is selected as a substitute; P3 is the chemical plant area in the evening. The first row of the algorithms selected for P1, P2, and P3 is from left to right, followed by low illumination images, AIRDANE, Demist enhancement algorithm, ISP, and LIME. The second row is from left to right, followed by Retinex, GLAD, BIMEF, StableLLVE, and the improved algorithm in this article.

As shown in Fig. 6, the first row of P1, P2, and P3 from left to right is low illumination image, AINDANE, Demist enhancement, ISP, and LIME, and the second row from left to right is Retinex, GLAD, BIMEF, StableLLVE and the improved algorithm. P1 is the factory workers working at night; due to the lack of a low-light data set of oil tankers, P2 selects the image of the vehicle in the LOL data set. The image contains multiple cars and buildings, and the feature information of the image is more complex than that of the simple oil tanker. P3 is a chemical plant under operation in the evening.

From the perspective of visual analysis^24^, different algorithms will produce different effects for different images. It can be seen from P1 that the image color distortion after AINDANE, ISP, and GLAD enhancement. The visual effect of LIME is better than that of BIMEF, StableLLVE, and Demist enhancement, but its enhanced image has too strong illumination. The image enhanced by the Retinex algorithm can see that the edge of the road surface is blurred, and the enhanced effect of this algorithm is ideal, which is in line with the expected results.

It can be seen from P2 that the effect of Demist enhancement and Retinex algorithm is poor, the overall image is darker, the color distribution of ISP and AINDANE is uneven, the image distortion is more serious, the image after LIME enhancement is too sharp, the saturation and contrast of GLAD and BIMEF images are too strong, resulting in the overall darkness of the image, and the color recovery of StableLLVE is poor. Compared with other images, there is an obvious color difference problem. The image enhanced by this algorithm is clearer, and the overall tone and detail recovery of the enhanced image are ideal.

The StableLLVE image in P3 contains too much noise and the image is blurred. The image sky after LIME enhancement is too bright, and the brightness enhancement effect of GLAD and Demist enhancement is not obvious. There is a color difference in the lower right corner image of AINDANE and BIMEF images, and there are artifacts on the edge of the ISP, which affects visual aesthetics. The algorithm in this paper is more perfect in detail and texture feature processing and the overall enhancement effect is better.

### Numerical analysis

The image quality evaluation indexes selected in this paper include PSNR (peak signal-to-noise ratio) and SSIM (structural similarity). Both of them are the most commonly used image evaluation indexes. The larger the value, the better. The specific calculation formula is:

8 $$\begin{aligned} PSNR = 10*\lg \left[ {\frac{{{{\left( {2^{M}} - 1\right) }^{2}}}}{{\frac{1}{{mn}}\sum \limits _{i = 0}^{m - 1} {\sum \limits _{j = 0}^{n - 1} {{{\left| {{S_{nor}}(i,j) - {S^{'}}(i,j)} \right| }^2}} } }}} \right] \end{aligned}$$

In the formula, m and n represent the height and width of the image, ${S_{nor}}$ is the normal illumination image, ${S^{'}}$ is the low illumination enhanced image, and M is the number of bits of the image pixels (usually 8 bits).

The calculation formula of SSIM is :

9 $$\begin{aligned} \begin{aligned} SSIM\left( {S_{nor}},{S^{'}}\right) =&\,\left( 2{\mu _{{S_{nor}}}}{\mu _{{S^{'}}}} + {c_1}\right) \left( 2{\sigma _{{S_{nor}}{S^{'}}}} + {c_2}\right) \\ {}&/\left( {\mu _{{S_{nor}}}^{2}} + {\mu _{{{S^{'}}}^2}} + {c_1}\right) \left( {\sigma _{{{S_{nor}}}^2}} + {\sigma _{{{S^{'}}}^2}} + {c_2}\right) \end{aligned} \end{aligned}$$

In the formula, ${\mu _{{S_{nor}}}}$ and ${\mu _{{S^{'}}}}$ represent the mean value of the image, ${\sigma _{{S_{nor}}}}$ and ${\sigma _{{S^{'}}}}$ represent the variance of the image, ${\sigma _{{S_{nor}}{S^{'}}}}$ is the covariance of the image, both ${c_1} = {k_1}L$ and ${c_2} = {k_2}L$ are stable constants, and L is the dynamic range of the pixel value. (In this paper, $k_1$= 0.01, $k_2$= 0.03)

Through the above description, it can be seen that both of them need to use the normal image as a reference. When the value is larger, it is proved that the distortion of the image is smaller, the image is closer to the reference image, and the image quality is higher.

**Figure 7:**
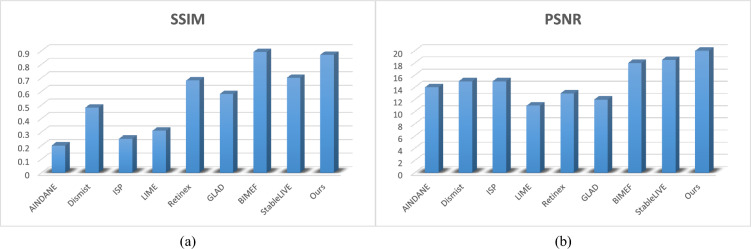
PSRN and SSIM values for enhanced results.

To evaluate the quality of image enhancement more objectively, the two most commonly used image quality evaluation indexes, PSNR and SSIM^25,26^, are used. Because different algorithms have different effects on different images, this paper takes the average values of PSNR and SSIM of P1, P2, and P3. The specific results are shown in Fig. 7.

It can be seen from the graph that the PSNR value of the improved Retinex algorithm is the largest of all algorithms. Its SSIM is located in second place, which is 0.01 different from the BIMEF algorithm but compared with other algorithms, it is in a relatively leading position.

In recent years, in image reconstruction tasks, higher PSNR and SSIM do not necessarily represent better image quality. Reference^27^ indicates that images with higher PSNR and SSIM do not necessarily have texture details that conform to human visual habits. Therefore, researchers have proposed a natural image quality evaluator–NIQE^27^. The biggest difference between NIQE and PSNR and SSIM is that NIQE is a non-reference image evaluation index, which no longer requires subjective evaluation scores for images, It extracts the features in the natural landscape to test the test image and constructs a multivariate Gaussian model to measure the difference in the multivariate distribution of the reconstructed image. As an indicator for evaluating real image restoration, the smaller the value, the better. To verify the feasibility of the algorithm, NIQE values were calculated using three categories of factory area images: people, vehicles, and factories. The specific data is shown in Table 3, and compared with the other eight algorithms:

**Table 3:** Comparison results of NIQE for images processed by different algorithms.

Images	Oringial	AINDANE	Demist	ISP	LIME	Retinex	GLAD	BIMEF	StableLLVE	Ours
People	5.12	4.64	3.43	5.07	4.83	5.14	4.12	5.55	5.54	**3.31**
Cars	4.53	5.19	4.55	5.46	5.28	4.05	5.31	4.38	**3.56**	3.86
Factory	4.89	4.08	3.72	5.39	4.31	3.74	4.82	4.24	3.74	**3.21**

The best-performing values are in bold.

A numerical analysis was conducted on the data in Table 3. The bold data in the table represents the minimum value of NIQE among several algorithms, which is also the algorithm with good image quality performance. It can be seen that the improved algorithm in this article has achieved good results in both People and Factory, ranking second in cars, second only to the StableLLVE algorithm, and overall performance is relatively excellent.

IE (information entropy) is a quantitative indicator of the image information content. The larger the value, the richer the image details are, and the better the image quality is:

10 $$\begin{aligned} IE = - \sum \limits _{r = 0}^R {P(r)\log [P(r)]} \end{aligned}$$

In the formula, *P*(*r*) is the probability of the image appearing at a certain gray level r in the image. To verify the algorithm’s control over image details, the specific values of IE are shown in Table 4:

**Table 4:** Comparison results of IE for images processed by different algorithms.

Images	Oringial	AINDANE	Demist	ISP	LIME	Retinex	GLAD	BIMEF	StableLLVE	Ours
People	6.23	7.55	6.02	7.51	5.82	7.31	7.42	7.1	7.23	**7.78**
Cars	6.84	7.75	7.32	7.88	7.42	7.44	7.82	7.84	7.49	**7.94**
Factory	7.5	6.66	7.37	7.8	7.28	7.83	7.74	7.28	7.33	**7.89**

The best-performing values are in bold.

Analyzing the data in Table 4, it can be seen that the algorithm in this paper achieved maximum values in both IE and did not ignore the information of the image during the enhancement process, resulting in better enhancement effects for low illumination images.

LOE reflects the natural preservation ability of the enhanced image, and the higher its value, the better the brightness order of the image, making it appear more natural. It is also an important indicator of low illumination image enhancement. Therefore, LOE is defined based on the brightness order error between the original image I and its enhanced image ${I_e}$. The brightness $L\left( {x,y} \right)$ of the image is the maximum value of its three color channels RGB:

11 $$\begin{aligned} L(x,y) = \mathop {\max }\limits _{c \in \{ r,g,b\} } {I^c}(x,y) \end{aligned}$$

For each pixel (x, y), the relative order difference in brightness between the original image I and the enhanced image ${I_e}$ is defined as:

12 $$\left\{ {\begin{array}{*{20}l} {RD(x,y) = \sum\limits_{{i = 1}}^{m} {\sum\limits_{{j = 1}}^{n} {U(L(x,y),L(i,j)){\text{ }}xor{\text{ }}U\left( {L_{e} (x,y),L_{e} (i,j)} \right)} } } \hfill \\ {U(x,y) = \left\{ {\begin{array}{*{20}l} {1,} \hfill & {\quad {\text{for }}x \ge y} \hfill \\ {0,} \hfill & {\quad {\text{else}}} \hfill \\ \end{array} } \right.} \hfill \\ \end{array} } \right.$$

Where m and n are the height and the width, *U*(*x*, *y*) is the unit step function, and *xor* is the exclusive-or operator. LOE is defined as:

13 $$\begin{aligned} LOE = \frac{1}{{m*n}}\sum \limits _{i = 1}^m {\sum \limits _{j = 1}^n {RD(i,j)} } \end{aligned}$$

In order to reduce the computational complexity, we take the down-sampled versions DL and $D{L_e}$ of size $dm*dn$ instead of L and ${L_e}$. The ratio r between the size of the down-sampled image and that of the original images is set as $r = 50/\min (m,n)$. As a result, the size $dm*dn$ of the down-sampled image is $(m*r)*(n*r)$.

The values of m and n in Eq. 13 are shown in Table 5.

**Table 5:** m and n parameter settings.

Images	m	n
People	50	50
Cars	50	56
Factory	75	50

To verify whether the image has a better brightness order, the specific values of its LOE are shown in Table 6:

**Table 6:** Comparison results of LOE for images processed by different algorithms.

Images	Oringial	AINDANE	Demist	ISP	LIME	Retinex	GLAD	BIMEF	StableLLVE	Ours
People	353.02	654.6	231.37	153.95	208.24	195.89	381.01	174.32	237.31	**71.59**
Cars	408.2	506.7	233.14	374.26	**171.6**	187.07	231.09	188.66	187.49	174.83
Factory	360.23	784.06	268.92	228.56	279.17	284.96	313.84	196.31	563.01	**147.24**

The best-performing values are in bold.

Analyzing the data in Table 6, it can be seen that the LOE value of the algorithm in this paper is the lowest in People and Factory, which is the most natural. In Cars, the LIME algorithm performs better than the algorithm in this paper but only 3.23 times worse. The overall performance of the algorithm in this paper is better.

In summary, the improved Retinex algorithm ranks first among the nine algorithms in both PSNR and IE metrics, while slightly lagging behind the BIMEF algorithm with a difference of 0.01 in SSIM metrics; In terms of NIQE indicators, it is second only to the StableLLVE algorithm, but far exceeds the BIMEF algorithm; In terms of LOE indicators, although Cars lag behind LIME, its average value in three categories of images is far greater than that of the algorithm. In general, the algorithm in this paper performs well on different objective indicators, which also confirms the feasibility, reliability, and superiority of the algorithm in this paper.

## Conclusion

In this paper, a low illumination enhancement model based on an improved Retinex algorithm is proposed. The model is divided into three modules. In the decomposition module, a USD-Net network structure is established, forming two branches: illumination and reflection for illumination enhancement and reflection denoising; In the illumination enhancement module, the Mobilenet-v3 lightweight network is introduced and the residual structure is used in the network to enhance the image feature extraction ability and simplify the network as much as possible. Aiming at the problem of noise influence encountered by the reflection module, the BM3D denoising method is adopted. Finally, the contrast-adjusted illumination component and the denoised reflection component are fused to obtain rich texture, clear edge, and good contrast. Experiments show that the improved algorithm proposed in this paper has a better enhancement effect than the other eight advanced algorithms, which can meet the needs of chemical plants.

This article draws the following conclusions on the improved model:

1. This article applies the BM3D method to image denoising in reflection reconstruction networks. Although this method has good denoising performance, the training time is too long. Therefore, in the future, we will conduct in-depth research on the denoising methods of BM3D to achieve better performance and denoising effects.
2. The improved Retinex algorithm in this article is only applicable to the processing of static low-illumination images and cannot be applied to dynamic low-illumination videos. In the future, it is planned to expand its application scope to the enhancement of low-illumination videos.

## Data Availability

The authors confirm that the data supporting the findings of this study are available within the article.
